# The Survival Rate of Living-Donor Liver Transplantation Between Same-Sex and Opposite-Sex Recipients

**DOI:** 10.3390/diagnostics15060757

**Published:** 2025-03-18

**Authors:** Mei-Yun Wu, Yu-Hung Lin, Wei-Juo Tzeng, Shih-Feng Weng, Wan-Ching Chang, Chich-Hsiu Hung

**Affiliations:** 1 School of Nursing, Kaohsiung Medical University, Kaohsiung 807, Taiwan; 123@cgmh.org.tw; 2 Department of Nursing, Kaohsiung Chang Gung Memorial Hospital and Chang Gung University College of Medicine, Kaohsiung 833, Taiwan; 3 Department of Surgery, Kaohsiung Chang Gung Memorial Hospital and Chang Gung University College of Medicine, Kaohsiung 833, Taiwan; adrianlin107@gmail.com (Y.-H.L.); jbkim22004@gmail.com (W.-J.T.); 4 Department of Health Care Administration and Medical Informatics, Kaohsiung Medical University, Kaohsiung 807, Taiwan; sfweng@kmu.edu.tw; 5 Department of Diagnostic Radiology, Kaohsiung Chang Gung Memorial Hospital and Chang Gung University College of Medicine, Kaohsiung 833, Taiwan; o927003551@gmail.com; 6 Department of Nursing, Asia University, No. 500, Lioufeng Rd., Wufeng District, Taichung 41354, Taiwan

**Keywords:** liver transplantation, living-donor liver transplantation, sex matching, survival rates

## Abstract

**Purpose**: Living-donor liver transplantation (LDLT) serves as a solution for patients facing end-stage liver disease. The existing literature indicates that sex differences between transplant donors and recipients might influence survival rates. **Methods**: We used a retrospective study design to investigate the impact of different sex pairings on the survival rates of adult LDLT recipients. This study involved the long-term tracking of recipients who underwent LDLT between 7 June 2000 and 31 December 2021. **Results**: In total, 169 pairs (37.1%) of male recipients with male donors, 145 pairs (31.8%) of male recipients with female donors, 77 pairs (16.9%) of female recipients with female donors, and 65 pairs (14.3%) of female recipients with male donors were submitted for analysis. With independent *t*-tests or chi-squared tests demonstrating that liver weight and graft-to-recipient weight ratio (GRWR) for same-sex LDLT recipients were significantly higher than opposite-sex recipients; significant differences in disease diagnoses between same-sex and opposite-sex LDLT recipients were found. The Kaplan–Meier survival curve indicates that while same-sex pair survival rates were higher than opposite-sex pairs, the difference was not statistically significant. **Conclusions**: While sex matching might have some impact on survival rates, it is influenced by a variety of factors, so the effects of donor and recipient sex matching on liver transplantation remains controversial. The findings of this study can serve as a reference for living-donor liver transplant teams when making donor selection decisions.

## 1. Introduction

Due to the shortage of available organs for transplantation, the development of living-donor liver transplantation (LDLT) can address the mortality crisis faced by patients with end-stage liver disease and liver cancer [1] while further solving the problem of insufficient organ donations [2,3,4]. Most transplant centers consider multiple factors regarding both recipients and donors and strive to improve post-transplant survival rates [5,6]. Factors affecting the survival of recipients include pre-transplant evaluations of both donors and recipients, the medical team’s expertise, and the quality of post-transplant critical care [5,7,8].

In the severity of the disease (model for end-stage liver disease: MELD Score) in end-stage liver disease patients [9,10], the presence of chronic disease history (diabetes, hypertension, chronic kidney disease), age, body mass index (BMI), hepatocellular carcinoma (HCC), and gender can affect the survival rate of the recipient [4,11,12]. In addition to the condition of the recipients, the graft-to-recipient weight ratio (GRWR) in terms of the ratio of donor liver volume to recipient weight, body mass index (BMI), and demographic characteristics of the donors [13] also affect the prognosis and survival rate of the transplantation, including age and gender [4,7,8]. Liver transplantation is a manifestation of cross-team care, where every crucial aspect is jointly managed, experiences are continually accumulated, and care models are improved through learning from these experiences [14,15]. For intensive care providers after transplantation surgery, besides maintaining the stability of the patient’s vital signs, the timely detection of signs of complications and providing appropriate care is the most important role in the care process [16].

The sex differences between the donor and recipient in transplantation can affect the survival rate of the cases [17,18]. These phenomena also exist in both nonliving and living kidney and liver transplantation surgeries [19]. Regarding the long-term survival rates of adults receiving LDLT, most studies focus on physiological indicators, transplantation techniques, and treatment-oriented aspects. Furthermore, most research designs are cross-sectional [4,10,20]; moreover, when exploring factors affecting patient survival rates, research results can be biased due to the use of different subjects from various institutions [4,11].

The sex of donors and recipients can affect the prognosis and survival [4,10]. Past studies, however, have shown significant variability in the number of subjects and lack long-term follow-up; accordingly, this study aimed to longitudinally analyze sex-matched survival rates.

## 2. Materials and Methods

### 2.1. Design

A retrospective study design was employed to investigate the impact of different sex pairings on the survival rates of adult LDLT, involving the long-term tracking of recipients who underwent LDLT from 7 June 2000 to 31 December 2021, with the follow-up extending to 31 December 2022.

### 2.2. Subjects

The subjects of this study were recipients aged 20–65 years, matched with inclusion criteria restricting eligible donors to fifth-degree relatives aged 20–50 years. The exclusion criteria involved (1) recipients with a MELD score of 25 or higher; (2) recipients with untreated hypertension, diabetes, or chronic kidney disease prior to transplantation; (3) recipients with a body mass index (BMI) of 30 or higher; (4) recipients not meeting the eligibility criteria of the University of California, San Francisco (UCSF), for hepatocellular carcinoma (HCC) and not having been treated for the tumor; (5) donors with a graft-to-recipient weight ratio (GRWR) of 0.8 or less; (6) donors with a body mass index (BMI) of 30 or higher; and (7) donors with 10% or higher fatty liver content or 5% or higher hepatic steatosis.

The sample size estimation for this study was conducted using the UCSF Clinical & Translational Science Institute’s Sample Size—Survival Analysis software, including both same-sex and opposite-sex living-donor liver transplant recipients (N = 456).

### 2.3. Research Instruments

The study data were collected using a retrospective chart review method at a medical center in southern Taiwan. The research subjects were recipients of adult living-donor liver transplants between 7 June 2000 and 31 December 2021, and their survival rates were tracked until 31 December 2022.

### 2.4. Research Ethics

The researchers obtained approval from the Chang Gung medical Foundation Institutional review board (IRB, NO 202300027B0; Date, 31 January 2023) of the enrolling institution before conducting the study. During the study, the privacy and confidentiality of all research subjects were ensured, and all data were handled in a completely de-identified and anonymous manner.

### 2.5. Data Analysis

#### 2.5.1. Descriptive Statistics

Continuous variables in the demographic data of the research subjects, including age, body mass index (BMI), model for end-stage liver disease (MELD) score, and the ratio of donated liver volume to the recipient’s weight, were analyzed using mean and standard deviation modalities. Categorical variables in the demographic data, including gender, education level, religious belief, marital status, occupation, presence of comorbidity (hypertension, diabetes, and kidney disease), disease diagnosis, and sex matching between donor and recipient, were presented using counts and percentages.

#### 2.5.2. Inferential Statistics

The cumulative survival rate was calculated using the Kaplan–Meier method, tracking from the liver transplantation until the occurrence of the event (death). Kaplan–Meier survival curves were plotted, and the Log-rank Test was used to examine whether there were statistically significant differences in survival curves between different groups.

## 3. Results

### 3.1. Demographic Characteristics

From 7 June 2000 to 31 December 2021, a total of 1748 individuals underwent LDLT. Among them, 456 participants met the study criteria, including 314 male recipients (68.9%) and 142 female recipients (31.1%), with a male-to-female ratio of 2:1. Ultimately, 169 male recipients received livers from male donors (37.1%); 145 male recipients received livers from female donors (31.8%); 77 female recipients received livers from female donors (16.9%); and 65 female recipients received livers from male donors (14.3%) for a total of 246 same-sex liver transplants (54%) and 210 opposite-sex liver transplants (46%).

The demographic characteristics of same-sex recipients and opposite-sex recipients are shown in Table 1. The two groups were analyzed separately using an independent sample *t*-test or chi-squared test, with the results revealing that liver weight and GRWR of the same-sex living-donor liver transplant recipients were higher than those of the opposite sex; furthermore, there was a significant difference in disease diagnoses between same-sex and opposite-sex living-donor liver transplant recipients (Table 1).

### 3.2. Survival Analysis

In total, 456 pairs of recipients met the inclusion criteria for LDLT. The cumulative survival rate of the entire cohort up to the present time (t) is calculated as the t-year survival probability, by sequentially multiplying the survival rates at each interval. These rates are 95.6% for 1 year, 92.6% for 3 years, 91.3% for 5 years, 85.6% for 10 years, 81% for 15 years, and 78.4% for 20 years. The cumulative number of deaths at each interval were 20 at one year, 33 at three years, 38 at five years, 54 at ten years, 60 at fifteen years, and 61 at twenty years. Consequently, the average survival time was 18.22 years, with a total of 61 deceased recipients (Table 2).

Among the recipients there were 246 same-sex pairs. The cumulative survival rate of the entire cohort up to the present time (t) represents the t-year survival probability calculated by sequentially multiplying the survival rates at each interval. These survival rates are 95.1% for 1 year, 93.3% for 3 years, 93.3% for 5 years, 88.3% for 10 years, 85.8% for 15 years, and 81.3% for 20 years. The cumulative number of deaths at each interval was 12 at one year, 16 at three years, 16 at five years, 24 at ten years, 26 at fifteen years, and 27 at twenty years. The average survival time was 18.75 years.

**Figure 1:**
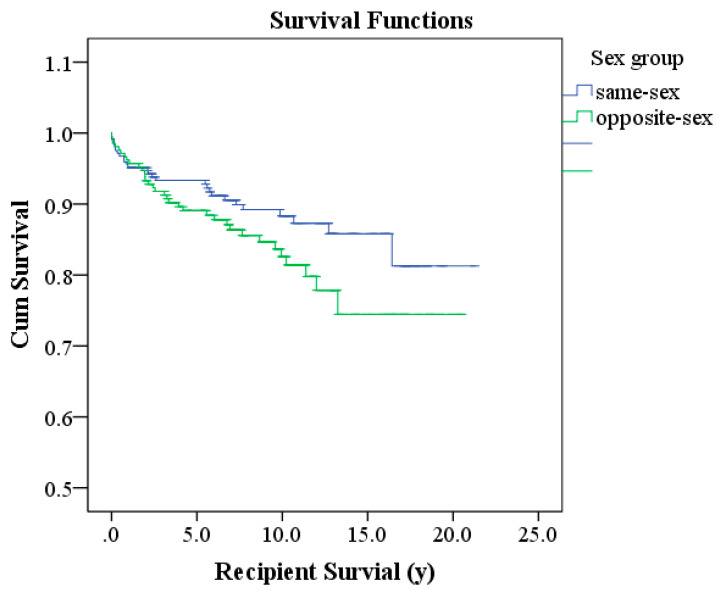
Kaplan–Meier method (*p* = 0.092).

There were 210 opposite-sex pairs, with a survival rate of 96.2% at 1 year, 91.8% at 3 years, 89.1% at 5 years, 82.6% at 10 years, 74.5% at 15 years, and 74.5% at 20 years. The cumulative number of deaths at each interval was 8 at one year, 17 at three years, 22 at five years, 30 at ten years, 34 at fifteen years, and 34 at twenty years. The average survival time was 16.97 years (Table 3). Although the survival rate for same-sex pairs was higher than that for opposite-sex pairs, the difference was not statistically significant (*p* = 0.092) (Table 3). The Kaplan–Meier survival curve shows that same-sex pairs had higher survival rates than opposite-sex pairs, but the difference was not substantial (Figure 1).

Multivariate analysis: Relative to the same sex (reference group), the risk ratio for the opposite sex is 1.828 (95% CI: 1.032–3.237), with a *p*-value of 0.039 (<0.05), indicating that the risk for the opposite-sex group is significantly higher in this sample. The combination of HBV + HCV has a significant impact in the multivariate analysis, with a risk ratio of 9.300 (95% CI: 2.397–36.086) and a very small *p*-value (<0.05), indicating that this combination significantly increases the risk of the event. The risk ratio for BMI <18.5 is 3.117 (95% CI: 1.087–8.941), which is significant in the univariate analysis (*p* = 0.034), indicating that a low BMI is associated with an increased risk of the event. The risk ratio for BMI >24 is 4.849 (95% CI: 1.829–12.854), showing a significant increase in risk in the multivariate analysis. In the multivariate analysis, for every 10-point increase in the MELD score, the risk ratio is 1.995 (95% CI: 1.126–3.535), indicating that liver dysfunction may significantly impact prognosis (Table 3).

Gender (opposite sex), diagnosis (HBV + HCV), height, BMI, and MELD score all show significant risk impacts. The noteworthy variables include BMI and the HBV + HCV combination, which have strong predictive power in the risk model. These results help us to further understand which factors significantly affect survival outcomes in both multivariate and univariate models (Table 3).

## 4. Discussion

The aim of this study involving the long-term follow-up of survival rates of sex-matched adult LDLT subjects was to examine the impact of sex matching between donors and recipients on patient survival rates after liver transplantation. This study tracked recipients of adult LDLT from the time of surgery until the occurrence of death and analyzed the survival rate over a defined period [21]. The empirical results of this study could serve as a reference for sex matching in LDLT, with the aim of improving the survival rates of recipients [22].

This study found that the survival rate for same-sex living-donor liver transplants was slightly higher than that for opposite-sex living-donor liver transplants, although the difference did not reach statistical significance. Whether sex differences are a risk factor for lower survival rates after LDLT remains inconclusive because study results can be influenced by various factors, such as sample size, study design, and the demographic characteristics of the study samples. Some studies have found that gender differences affect transplant outcomes; for instance, female recipients in male donor transplant groups have the lowest recurrence rate of liver cancer [21], but conversely, when female donors are paired with male recipients, it can have adverse effects on the transplanted liver [22].

Accordingly, sex matching should not be the sole consideration for doctors and patients when making decisions about liver transplantation. Each patient’s situation is unique, and treatment plans should be customized based on the individual’s needs and characteristics. Sex might have some impact on the survival rate of liver transplants, but this is influenced by various factors, including the patient’s overall health, the surgical process, immune response, post-transplant care, and patient compliance.

Males and females have different physiological structures and functions, which can also affect the progress and recovery of surgery. For example, differences in weight, height, and liver size between males and females could impact the difficulty of surgery and the success rate of transplantation. Some studies have found that estrogen might influence transplant outcomes [23], while there could also be differences between males and females in lifestyle, healthcare needs, and other aspects. These findings suggest that alterations in the sex hormone milieu or differences in estrogen and androgen receptor expression within the graft may contribute to variations in graft failure rates [24] further demonstrated that grafts from female donors transplanted into male recipients were associated with reduced survival rates, though the clinical significance of this observation remains uncertain. More recently, [25] reported an increased risk of graft failure specifically in female-to-male liver transplants, possibly attributed to a sharp decline in hepatic estrogen levels. Notably, estrogen levels in the female liver tend to decrease with age (post-40 years) or in cases of liver injury, which may partly explain poorer transplant outcomes in such cases.

### Limitation Paragraph

This study could benefit from further analysis by disaggregating donation data based on gender, such as male-to-female and female-to-male donations. In addition, when analyzing survival rates, it would be valuable to not only categorize participants by same-gender and different-gender groups but also to investigate the underlying reasons for higher mortality rates in certain genders. A more detailed explanation of these factors would enhance the findings. The results of this study could provide a reference for living-donor liver transplant teams when making decisions regarding donors, although as it reflects the results of a single transplant center, more related research and discussion in the future should be undertaken.

## 5. Conclusions

In summary, whether sex matching between living donors and recipients affects liver transplantation is a subject still open to debate.

## Figures and Tables

**Table 1:** Demographic characteristics of same-sex and opposite-sex living-donor liver transplant recipients (N = 456).

Demographic	Same Sex N = 246 (54%)	Opposite Sex N = 210 (46%)	*p* Value
Age	54.41 ± 6.92	53.14 ± 8.42	0.08
Height	163.53 ± 8.18	163.06 ± 7.61	0.53
Body weight	64.70 ± 9.65	63.31 ± 10.69	0.14
BMI	24.13 ± 2.65	23.74 ± 3.24	0.17
MELD	12.67 ± 4.98	12.81 ± 4.68	0.76
Graft weight	698.46 ± 134.48	651.96 ± 133.78	0.0001
GRWR	1.08 ± 0.20	1.03 ± 0.19	0.01
Diagnosis			0.01
HBV	54 (22.0%)	49 (23.3%)	
HCV	58 (23.6%)	28 (13.3%)	
HBV + HCV	3 (1.2%)	5 (2.4%)	
Alcoholic	25 (10.2%)	37 (17.6%)	
PBC/Cryptogenic/Autoimmune	16 (6.5%)	26 (12.4%)	
HBV + HCC	51 (20.7%)	30 (14.3%)	
HCV + HCC	24 (9.8%)	17 (8.1%)	
Other	15 (6.0%)	18 (8.5%)	

BMI: body mass index; GRWR: graft-to-recipient weight ratio; HBV: Hepatitis B virus; HCC: hepatocellular carcinoma; HCV: Hepatitis C virus; MELD: model for end-stage liver disease, MELD score; PBC: Primary Biliary Cirrhosis.

**Table 2:** Recipient survival rates at 1 year, 3 years, 5 years, 10 years, 15 years, and 20 years (N = 456).

Recipient	Death	Survival Rates (Years)	1 Year	3 Years	5 Years	10 Years	15 Years	20 Years
456	61	18.22	95.6	92.6	91.3	85.6	81	78.4
death/survival			20/436	33/388	38/343	54/175	60/65	61/31

**Table 3:** Cox regression of same-sex and opposite-sex living-donor liver transplant recipients (N = 456).

Risk Factor	Univariate Analysis		Multivariate Analysis	
	*p* Value	Hazard Ratio (95.0% CI)	*p* Value	Hazard Ratio (95.0% CI)
Sex group				
Same sex	Ref.			
Opposite sex	0.101	1.528 (0.921–2.534)	0.039	1.828 (1.032–3.237)
Education level				
>College	Ref.			
High school	0.350	0.706 (0.341–1.164)	0.775	0.891 (0.405–1.959)
Secondary school	0.524	0.789 (0.381–1.636)	0.973	0.986 (0.436–2.230)
Primary school	0.788	0.910 (0.458–1.806)	0.546	1.309 (0.547–3.134)
Employment				
Fulltime	Ref.			
Unemployed/ Part-time	0.565	1.16 (0.700–1.924)	0.209	1.443 (0.814–2.556)
Religion				
None	Ref.			
Other	0.231	1.631 (0.733–3.632)	0.146	1.891 (0.800–4.467)
Taoism	0.494	0.806 (0.435–1.495)	0.355	0.728 (0.371–1.428)
Buddhism	0.398	0.723 (0.314–1.533)	0.518	0.776 (0.359–1.675)
Marital Status				
Married	Ref.			
Unmarried	0.923	1.059 (0.331–3.388)	0.627	0.705 (0.172–2.888))
Divorced/Widowed	0.358	0.516 (0.126–2.116)	0.256	0.419 (0.093–1.878)
Diagnosis				
HBV	Ref.			
HCV	0.308	1.493 (0.690–3.231)	0.07	2.182 (0.937–5.078)
HBV + HCV	0.958	1.056 (0.137–8.131)	0.989	0.985 (0.123–7.892)
Alcoholic	0.773	1.148 (0.451–2.924)	0.990	1.006 (0.384–2.638)
PBC/ Cryptogenic/ Autoimmune	0.783	0.853 (0.275–2.647)	0.576	0.707 (0.210–2.383)
HBV + HCC	0.293	1.514 (0.699–3.277)	0.076	2.106 (0.925–4.797)
HCV + HCC	0.900	0.930 (0.300–2.886)	0.781	1.181 (0.367–3.800)
Alcoholic + HCC	0.992	1.011 (0.131–7.832)	0.898	1.147 (0.141–9.355)
HBV + HCV + HCC	0.027	4.168 (1.173–14.810)	0.001	9.300 (2.397–36.086)
Other (Liver cyst P.S.C, IHD stone, Wilson’s disease, B.A Liver AVM, Wilson’s disease, Poly cystic, HCC, Hemangioma)	0.981	0.976 (0.127–7.521)	0.533	0.459 (0.040–5.310)
BMI				
18.5–24	Ref.			
<18.5	0.034	3.117 (1.087–8.941)	0.952	1.046 (0.242–4.517)
>24	0.376	1.266 (0.751–2.132)	0.002	4.849 (1.829–12.854)
MELD/10	0.158	1.447 (0.867–2.416)	0.018	1.995 (1.126–3.535)
Graft weight (gm)/10	0.758	1.003 (0.983–1.023)	0.450	1.010 (0.985–1.035)
GRWR > 1	0.063	1.210 (0.990–1.479)	0.892	1.049 (0.524–2.103)

BMI: body mass index; GRWR: graft-to-recipient weight ratio; HBV: Hepatitis B virus; HCC: Hepatocellular carcinoma; HCV: Hepatitis C virus; MELD: model for end-stage liver disease, MELD score; PBC: Primary Biliary Cirrhosis.

## Data Availability

The original contributions presented in this study are included in the article material. Further inquiries can be directed to the corresponding author(s).

## Abbreviations

The following abbreviations are used in this manuscript:

BMI	Body mass index
GRWR	Graft-to-recipient weight ratio
HBV	Hepatitis B virus
HCC	Hepatocellular carcinoma
HCV	Hepatitis C virus
HR	Hazard ratio
LDLT	Living-donor liver transplantation
MELD	Model for end-stage liver disease (MELD score)
PBC	Primary Biliary Cirrhosis
